# Testing the effectiveness of ecolabels to reduce the environmental impact of food purchases in worksite cafeterias: A randomised controlled trial

**DOI:** 10.1016/j.appet.2022.106277

**Published:** 2022-08-20

**Authors:** Rachel Pechey, Paul A. Bateman, Brian Cook, Christina Potter, Michael Clark, Cristina Stewart, Carmen Piernas, Susan A. Jebb

**Affiliations:** a Nuffield Department of Primary Care Health Sciences, Radcliffe Observatory Quarter, University of Oxford, Oxford, OX2 6GG, UK; b Oxford Martin Programme on the Future of Food and Nuffield Department of Population Health, University of Oxford, Oxford, OX3 7LF, UK; c Interdisciplinary Centre of Conservation Science, University of Oxford, 11a Mansfield Rd, OX1 3SZ, UK

## 1 Introduction

Meeting global climate targets will require a marked reduction in environmental impacts caused by dietary patterns (Willett et al., 2019), with several UK supermarkets setting targets to halve the environmental impact of customers’ food shopping by 2030 (Lee, 2021). The environmental impacts of different types of foods are highly variable, and variation in impact is also seen for a given food. For example, there is a 50-fold variability in the land-use impacts of beef products, although the difference in impacts of a given food (e.g. beef) is typically smaller than the difference in impacts between food types (e.g. beef vs beans) (Poore & Nemecek, 2018). For consumers to be able to make ecologically informed purchases, they need relevant information about the environmental impact of individual products at the point of choice.

A recent systematic review found that ecolabels (including a broad range of designs) are effective at promoting the selection, purchase, and consumption of lower environmental impact food and drink products (Potter et al., 2021). Since then, a series of studies using an experimental virtual online supermarket platform tested six different ecolabel versions (Potter, Pechey, Clark, et al., 2022; Potter, Pechey, Cook, et al., 2022). Across these studies, results indicated that providing a single environmental impact score (A-E) was effective at decreasing the environmental impact of participants’ food purchases. However, these studies were conducted in a hypothetical context, where participants did not spend any money nor receive the food they selected for purchase, and there remains a need to examine the effectiveness of such labels in real-world settings.

There is limited evidence on the role of ecolabels in experimental field trials, with the systematic review (Potter et al., 2021) identifying only three: two in stores (Elofsson, Bengtsson, Matsdotter, & Arntyr, 2016; Vlaeminck, Jiang, & Vranken, 2014), and one in a university cafeteria (Brunner, Kurz, Bryngelsson, & Hedenus, 2018). Cafeteria settings offer food for immediate consumption, with additional sensory cues such as aroma, as well as a different social context, which may alter responsiveness to labels compared to grocery stores (Bittner & Kulesz, 2015; McCrickerd & Forde, 2016). Previous research in university cafeterias may not offer a representative study setting. Worksite cafeterias are more likely to cater for a cross-section of the adult population, many of whom may not be specifically engaged in health promotion activities (Velema, Vyth, & Steenhuis, 2017), and the majority of employees eat at least one meal during their workday (Wunsch, 2018). The current randomised controlled trial tests the effectiveness of implementing eco-labelling to reduce the environmental impact of purchases in worksite cafeterias catering to a broad range of customers.

## 2 Methods

### 2.1 Design

This was a randomised controlled trial, with worksite cafeterias allocated to either an ecolabel or control (no label) condition using stratified randomisation (based on the number of meals sold per week). Randomisation was performed by a statistician allocating a list of site names using random numbers generated using STATA (Stata Statistical Software: Release 14. College Station, TX: StataCorp LP). Between 11^th^ May 2021 and 3^rd^ September 2021, sites that were randomised to the intervention group were asked to place ecolabels on their printed menus that were displayed at the point of meal selection.

Ethics approval was granted 02/12/2020 by the Central University Research Ethics Committee, University of Oxford (Ref: R72710/RE001). The study was pre-registered prospectively on ISRCTN (https://doi.org/10.1186/ISRCTN11266548; 7^th^ April 2021) and the Open Science Framework (https://osf.io/h2en3/; April 29, 2021).

### 2.2 Sites

**Fig. 1:**
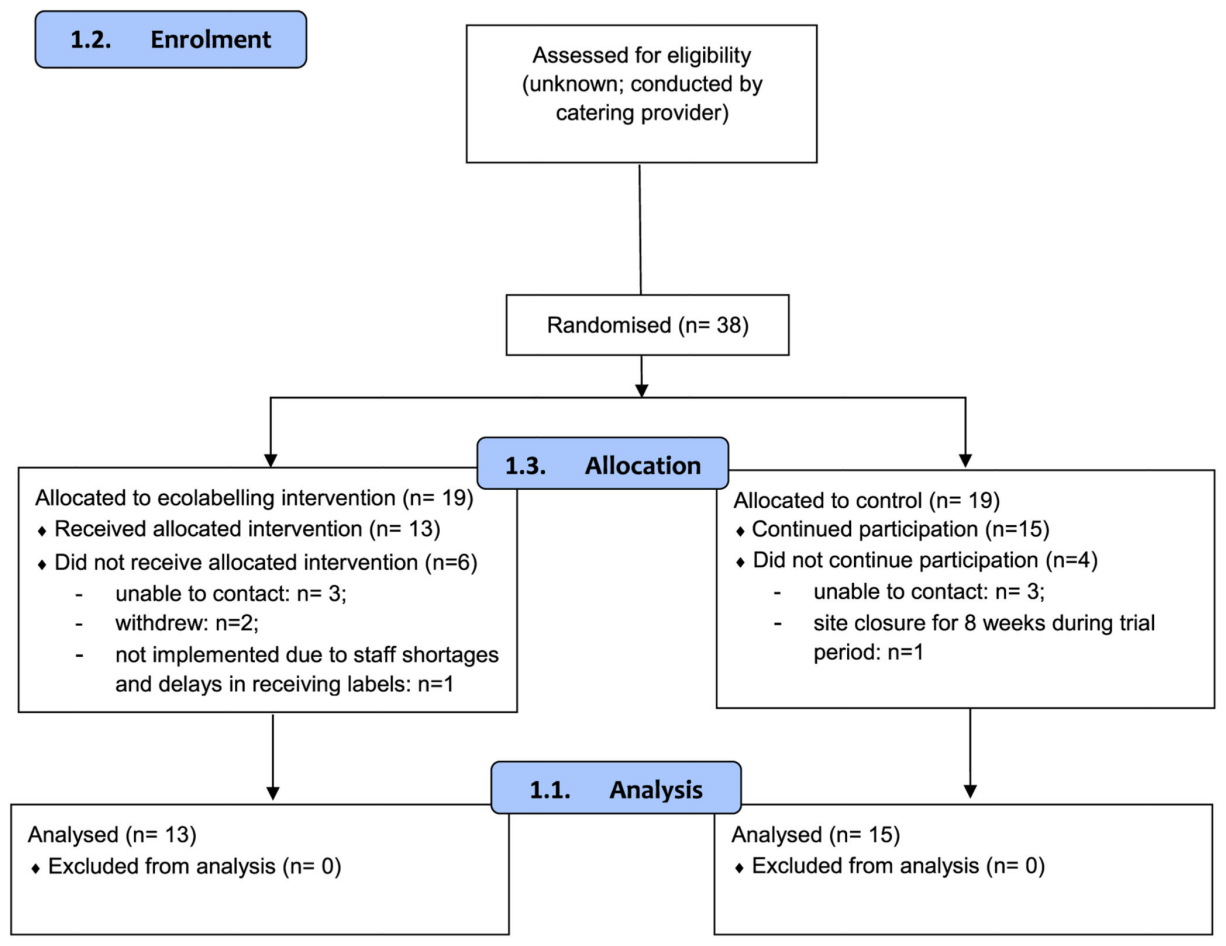
CONSORT 2010 flow diagram.

Working with a nationwide catering provider, 38 of their sites were identified (see Fig. 1). The inclusion criteria for the study specified that participating sites must be UK-based worksite cafeterias that had electronic point-of-sale tills hosted by the catering provider, and able to provide data at a detailed enough level to identify specific meals sold. All site cafeterias catered to staff working at manufacturing or distribution centres. Sites changed their main meals daily, with a 4-week menu cycle.

### 2.3 Intervention

The ecolabel values were generated based on ingredient-level data obtained from the catering provider for each hot menu item sold (see Supplementary Materials: Appendix A and Clark et al. (2022) for more information on label value calculations). The design was informed by formative research with members of the public and prior testing of a range of options (Potter, Pechey, Clark, et al., 2022; Potter, Pechey, Cook, et al., 2022).

**Fig. 2:**
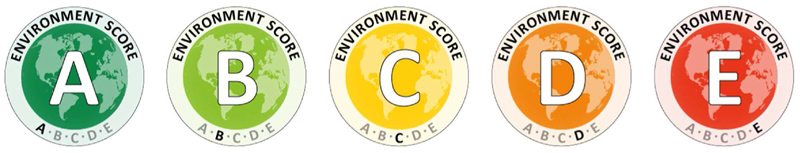
Ecolabels used in the trial.

**Fig. 3:**
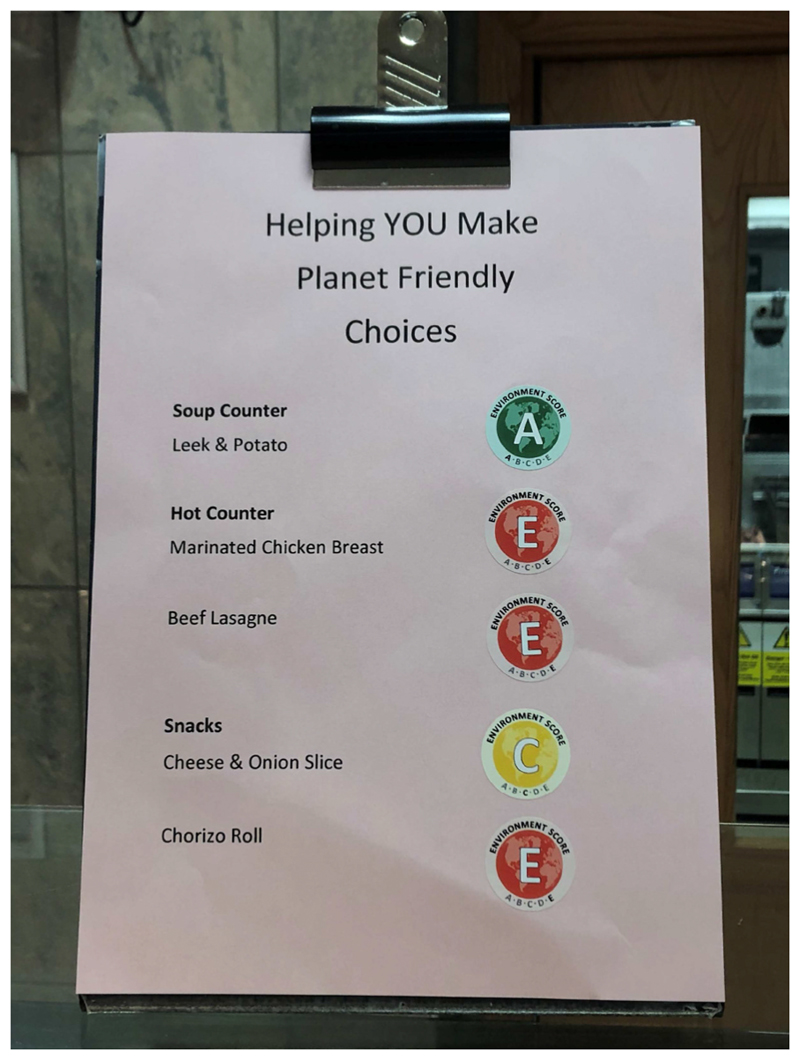
Example of ecolabels displayed on menus.

Ecolabel stickers (see Fig. 2) were prominently placed next to the name of each hot meal listed on the printed menus at each of the intervention sites during the intervention period (see Fig. 3). Menus were placed on top of food counters, so were present at the point of choice. Labels show environmental impacts as one of 5 letters (A-E), each with its own colour (from dark green to dark red) representing the lowest to highest environmental impact, analogous to energy-rating schemes.

### 2.4 Procedure

The research team generated environmental impact scores for each hot meal option by linking the ingredients in each recipe to environmental databases (Poore & Nemecek, 2018). The absolute impacts of meals were calculated across four environmental indicators (greenhouse gas emissions, land use, scarcity weighted water use, and eutrophication potential), which were then condensed into a single overall environmental impact score by placing equal weight on each indicator. This single environmental impact score was used to categorise meals into an A (lowest impact, most sustainable) to E (highest impact, least sustainable) scale. The full portfolio of possible meals (i.e. across all categories such as soups, jacket potatoes and hot main meals) at all sites operated by the provider, were evenly allocated to one of the five eco-label scores (i.e. 20% of meals were categorised into each of the A-E scores).

The meals comprised hot main meals, jacket potatoes, hot sandwiches (e.g. panini, toasties), hot savoury snacks (e.g. sausage rolls, pasties), and starters (e.g. soup). Ecolabel stickers (Fig. 2) were sent to each intervention site, and cafeteria staff were asked to add these to printed menus each day during the intervention period. Sites also printed and displayed information sheets (either on cafeteria noticeboards or alongside their menus) giving some background to explain the ecolabels (see Supplementary Materials: Appendix B). Cafeteria users in both intervention and control cafeterias were not explicitly made aware of the research study during the trial period, though the intervention was, by necessity, unblinded.

Data on product-level sales at each site were obtained via the catering provider for the period from 1^st^ February 2021 to 3^rd^ September 2021. Data prior to 11th May comprised the baseline period in analyses.

#### 2.4.1 Fidelity checks

Researchers carried out visits to cafeterias to monitor the implementation of the ecolabels. One site was unable to be visited due to restrictions as a result of the Covid-19 pandemic. Concerns were raised about the implementation of the intervention during a visit in one instance, following which a researcher contacted the site manager to ensure these issues were addressed.

Given restrictions on the number of visits possible (one per site) due to distance and Covid-19 precautions, sites were also asked to regularly send photos of their menus, with the labels attached, and multiple phone calls were made to the managers of each site during the intervention period to ensure that the trial was running smoothly. One site received a second visit, given photos were unable to be sent partway through the trial, as the manager no longer had access to a suitable camera/phone.

Evidence collected on fidelity to protocol and site closures was used to create two site grouping variables to use in sensitivity analyses. The first grouping split sites according to whether we had regular evidence of fidelity (6 or more photos; plus site visit). The second grouping split sites according to whether we had evidence of fidelity (i.e. photos or calls confirming the presence of ecolabel stickers on menus) at the start and end of the trial.

### 2.5 Statistical analysis

The primary outcome was the environmental impact of purchases (EcoScore), as measured by the mean environmental impact score for products purchased from labelled categories across a week in each worksite cafeteria. A score of 1 represents an option with the lowest environmental impact, while a score of 100 represents options with the highest environmental impact. Weekend and bank holiday sales when transactions were expected to differ from the norm were excluded a priori. The outcome was calculated from sales data recorded via electronic point–of-sale tills through the period of the trial, combined with data on the environmental impact of each food option.

Analyses of secondary outcomes were planned to be conducted if the primary outcome showed an effect. These comprised: (1) Impact on total weekly revenue (£GBP) from each cafeteria; (2) Impact on the total number of transactions per week in each cafeteria; (3) Impact on total energy (kcal) and nutrient content purchased weekly from labelled categories in each worksite cafeteria (controlling for the total number of transactions).

Mixed effects modelling was used to analyse the primary outcome, which was normally distributed. Predictors included allocated group (control or intervention), a dummy variable for the trial period (baseline vs. intervention), the trial week number, the number of transactions per week, and a dummy variable to indicate weeks with bank holidays. The trial site was controlled for as a random effect. The interaction term between the allocated group and trial period variable was used to measure the intention-to-treat (ITT) effect.

When issues with fidelity in some sites became apparent during the conduct of the trial an alternative (not pre-registered) as-treated analysis was planned to examine the impact of ecolabelling in sites only when the ecolabelling was actually implemented, rather than when it was scheduled to start. Predictors were identical to the ITT analysis, with the exception of the trial period variable, which was omitted. Instead, an intervention implementation variable measured effectiveness in this analysis.

Significance was determined at the level of p < 0.05 for both the ITT and as-treated analyses.

Planned analyses of secondary outcomes followed the methods of the primary outcome analysis, using mixed effects modelling adjusting for weekly transactions (with the exception of the analysis examining transactions themselves) and study week, with site as a random effect.

All analyses were performed using STATA (Stata Statistical Software: Release 14. College Station, TX: StataCorp LP).

## 3 Results

Of the 38 sites randomised, 28 sites (13 intervention and 15 control) completed the study (see Fig. 1), with 11 of the 19 stratified randomisation pairings remaining complete. Dropouts occurred due to researchers being unable to contact site managers, sites withdrawing from the study (e.g. due to staff shortages), and prolonged site closures during the intervention period as a consequence of the Covid-19 pandemic. In addition, some sites had to close temporarily during the intervention due to issues with staffing (e.g. due to cafeteria staff self-isolating); in other cases, labelling was temporarily suspended while managers were absent. These violations from protocol were recorded by researchers during the fidelity check procedures and were taken into account during as-treated analyses.

A total of 111,837 hot meal options were sold during the 31-week period between February and September, split across 15 control sites and 13 intervention sites. The intervention trial period began at the start of week 15. The mean weekly EcoScore of meals purchased at baseline was 67.9 (s.d.10.9) for the control sites and 70.3 (s.d. 8.6) for the intervention sites. The mean weekly EcoScore of meals purchased during the intervention period was 69.9 (s.d. 9.0) for control sites and 71.3 (s.d. 8.4) for intervention sites (Supplementary Figs. S1a and S1b show mean weekly EcoScores by site). There were delays in implementing labels in several sites, and so the mean weekly EcoScore during label implementation was also calculated: 71.4 (s.d. 8.6) (Table 1).

### 3.1 The effect of ecolabels on weekly mean EcoScore

The mixed effects model for the ITT analysis of the mean weekly EcoScores showed no evidence of an impact of ecolabels, with no significant difference in the change between baseline and intervention period in intervention vs. control sites (coef. = −1.01, 95%CI -3.11 to 1.08, p = 0.34). There was no evidence of a difference in the environmental impact of purchases between the intervention group compared with the control at baseline (coef. = 2.17, 95%CI -1.63 to 5.97, p = 0.26), or during the intervention period compared with baseline period for the control stores (coef. = 0.87, 95%CI -1.48 to 3.23, p = 0.47) (Table 2).

The as-treated analysis also found no evidence of an impact of ecolabels on EcoScore (coefficient for intervention implementation = −0.90, 95%CI -2.81 to 1.01, p = 0.36) (Table 2).

Sensitivity analyses including (a) only those sites known to have the highest fidelity and (b) excluding sites known to have the lowest fidelity showed similar results (see Supplementary Table S2).

Exploratory analyses examined purchases by label value. Regardless of site group at baseline or intervention period, around 50% of the mean weekly meals sold had an EcoScore that qualified for an E-rated ecolabel (Table 3). A similar pattern of results was seen when looking at the meals available (rather than sold) by site by ecolabel value (See Supplementary Table S3). Meals rated A or D accounted for the next highest mean weekly sales (between 12 and 23%), while meals rated B accounted for the lowest percentage of sales (1–4%). Hot main meals accounted for 67% of all sales, of these more than 80% would have qualified for an ecolabel of D or E. The majority of hot sandwiches (93%) and savoury snacks (63%) had an ecolabel rating of E. In contrast, more than 70% of starters and jacket potatoes had an ecolabel of A (Table 4).

## 4 Discussion

This large trial conducted in 28 worksite cafeterias found no evidence of a change in the environmental impact of customers’ food purchases as a consequence of ecolabelling of meals displayed on menus.

The study took place in a worksite setting, examining actual food purchases, with an intervention period lasting almost four months, and included a larger number of sites than many previous studies of workplace food purchasing (Brunner et al., 2018; Garnett, Balmford, Sandbrook, Pilling, & Marteau, 2019; Reynolds et al., 2021). We used ecolabels developed with public involvement and selected based on the evidence of effectiveness in previous experimental studies (Potter,

Pechey, Clark, et al., pre-print; Potter, Pechey, Cook, et al., pre-print). We made quantitative estimates of EcoScores based on recipes for each possible meal option, taking into account impacts on greenhouse gas emissions, land use, scarcity weighted water use, and eutrophication potential. These scores are not perfect but provide the basis for a robust proof of concept study. Limitations of the study, however, include that labels were placed on menus rather than directly next to each option, and while the visibility or salience of these labels may be less than labels on packages, this does reflect the most feasible location in these real-world contexts for labelling meal options in a typical worksite cafeteria. This was conducted while restrictions were still in place due to the Covid-19 pandemic, which meant that there were fluctuations in customer and staffing levels, leading to some temporary site closures during the study, and some sites offering a more restricted menu. While we conducted as-treated analyses to explore the potential impact of deviations from protocol, we were not able to control for possible confounders that may impact both adherence and the primary outcome (environmental impact).

In the light of the Covid-19 restrictions and government advice to work from home where possible, the study only included cafeterias catering to staff at manufacturing and distribution centres. EcoScores were calculated for the full range of possible hot meal options that could be offered by the provider and divided into quintiles. Post-hoc it was apparent that the distribution of EcoScores in the meals offered at the study sites was highly skewed towards high environmental impact options. Around half of all meals sold were rated ‘E’ and evidence from the fidelity check photographs shows some instances where all the hot main meal options available were rated ‘E’, or otherwise offering minimal choice. It is possible that ecolabels may be more effective in contexts where there is a greater range of options available for each type of meal. Not only does this make the choice meaningful in terms of environmental impact, but the choice available may reflect established preferences of cafeteria patrons (Drewnowski, 1997).

It is also possible that the lack of effectiveness reflects the demographic profile (e.g. socioeconomic position, gender) of the workforce in these manufacturing (mostly blue-collar) workplaces, relative to the more general population samples in our previous online studies or in trials in university cafeterias. Further studies are needed to explore the impact of ecolabels among customers across a range of socioeconomic positions to specifically examine this potential moderator of effectiveness (Sarink et al., 2016). A review of the differential impact by socioeconomic position of different types of dietary interventions – based on limited evidence – suggested information-based interventions targeting individuals tend to differentially improve diets of those with higher socioeconomic positions (McGill et al., 2015). A systematic review of ecolabelling studies (Potter et al., 2021) found mixed evidence of the differential impact of ecolabels by income or education, albeit with more studies suggesting a greater impact of labelling for those with higher socioeconomic positions than vice versa (income: 7 studies suggesting greater impact for higher income, 2 suggesting greater impact for lower income, 3 no difference by income; education: 4 studies suggesting greater impact for higher educated participants, 1 suggesting greater impact for lower educated participants, 4 no difference by education).

Although there was no evidence from this study of an effect of eco-labels on consumer purchases, labelling may have effects elsewhere in the food system which were not directly assessed here. Communications with site managers during the study suggested that some were sometimes adjusting their menu to ensure that not all the main meal options on a particular day were rated ‘E’. However, differences in meals available by ecolabel value were not apparent between intervention and control sites, suggesting this was not happening systematically or on a broad enough scale for impact to be observed. That said, following the study, the catering provider also revised their menus to provide a greater variety of lower environmental impact products. It is possible that such changes to the relative availability of low environmental impact meals could have a larger impact on the sustainability of customer purchases in the future than the direct impact of labelling, as has been suggested for nutritional labelling (Adams, Mytton, White, & Monsivais, 2016; Capewell & Graham, 2010; Marteau, Ogilvie, Roland, Suhrcke, & Kelly, 2011; Marty, Cook, Piernas, Jebb, & Robinson, 2020). Future research needs to consider the impact of eco-labelling on the supply of food as well as the choices of consumers.

## Conclusion

In this study of worksite cafeterias catering to manufacturing and distribution centres, we found no evidence that ecolabels influenced the sustainability of food purchases. There is a continued need to test labels’ effectiveness in other real-world contexts, for example, where available options reflect a wider range of more vs. less sustainable options across each type of meal, and in more diverse workplaces. There is also a need to evaluate the wider impact on food provision, rather than just focusing on consumer demand, given that the introduction of ecolabels may increase the availability of lower environmental impact options in the longer-term.

## Supplementary Material

Supplementary data to this article can be found online at https://doi.org/10.1016/j.appet.2022.106277.

Supplementary information

## Figures and Tables

**Table 1:** Characteristics of key variables at control and intervention sites (Mean (s.d.)).

	Control sites	Intervention sites
Number of daily meal options	7.5 (3.6)	6.7 (3.8)
Number of daily “main meal” options	3.3 (2.2)	3.2 (2.7)
Weekly meals sold at baseline	158.2 (99.3)	142.7 (134.1)
Weekly meals sold during intervention period	129.8 (83.8)	116.1 (105.2)
Weekly EcoScore at baseline	67.9 (10.9)	69.9 (9.0)
Weekly EcoScore during intervention period	70.3 (8.6)	71.3 (8.4)
Weekly EcoScore while labels present	–	71.4 (8.6)

**Table 2:** Results of regression analyses predicting mean weekly EcoScore of meals sold, for intention-to-treat vs. as treated analyses.

	Intention to treat			As treated	
	Coefficients (95%CIs)	p-value		Coefficients (95%CIs)	p-value
Intervention group [ref: Control]	2.17 (–1.63, 5.97)	0.263		2.03 (–1.69, 5.74)	0.286
Intervention period [ref: Baseline]	0.87 (–1.48, 3.23)	0.468		Omitted	–
Intervention group * Intervention period	–1.01 (–3.11, 1.08)	0.344		Omitted	–
Intervention implemented [ref: No]	Omitted	–		- 0.90 (-2.81, 1.01)	0.357
Week number	0.13 (0.01, 0.25)	0.028		0.17 (0.10, 0.25)	<0.001
Bank holiday week [ref: No]	0.39 (–1.13, 1.91)	0.614		0.24 (–1.23, 1.70)	0.750
Weekly transactions	0.01 (0.00, 0.003)	0.036		0.01 (0.00, 0.03)	0.039
Constant	64.57 (61.06, 68.07)	<0.001		64.47 (60.96, 67.98)	<0.001
N	*863 observations from 28 cafeterias*			*863 observations from 28 cafeterias*	

**Table 3:** Percentage of meal sales by label value, by trial period and group.

Label	Control sites			Intervention sites	
	Baseline period	Intervention period		Baseline period	Intervention period
A	15.3	20.2		22.8	22.4
B	2.8	1.6		3.9	1.4
C	12.0	9.3		10.0	9.1
D	15.7	19.8		12.2	12.3
E	54.2	49.2		51.1	54.8
Difference between highest and lowest labels available daily ^a^	3.6 (1.0)	3.4 (1.2)		3.5 (1.2)	3.2 (1.4)

a Where a difference of 1 indicates e.g. a highest value of A and lowest of B, and a difference of 4 a highest value of A and lowest of E.

**Table 4:** Percentage sold by label value for meal type sold.

Label	Meal type						Total
	Jacket Potatoes	Main Meals	Hot Sandwiches	Savoury Snacks	Starters		
A	71.0	10.2	0.0	6.9	70.2		15.8
B	0.0	1.4	0.4	3.7	19.3		2.0
C	23.6	5.6	0.2	20.6	7.7		10.0
D	2.1	22.0	6.8	5.8	0.0		16.2
E	3.3	60.8	92.6	63.0	2.8		56.0

## Data Availability

The authors do not have permission to share data.

## Availability of data

The dataset comprises commercially sensitive data so is not publicly available.
