# Supplementary material for: Testing the effectiveness of ecolabels to reduce the environmental impact of food purchases in worksite cafeterias: A randomised controlled trial
Source: Appetite. Author manuscript; Available in PMC 2025 Nov 28. (PMC7618413; doi:10.1016/j.appet.2022.106277)
Supplement: Supplementary information [file EMS211092-supplement-Supplementary_information.docx]

**Testing the effect of ecolabels on the environmental impact of food purchases in worksite cafeterias: A randomised controlled trial**

**Supplementary Materials**

**Appendix A:** Calculation of ecolabel scores

**Appendix B:** Poster displayed at worksites explaining ecolabels

**Figures S1a and S1b**. Mean weekly EcoScore by site, for intervention sites (Figure S1a) and control sites (Figure S1b)

**Table S2**. Regression coefficients (95%CIs) predicting mean weekly EcoScore in per protocol analyses: Including only sites with the highest fidelity vs. excluding sites with the lowest fidelity

**Table S3**. Mean percentage of meal options available each day by label value

### **APPENDIX A: CALCULATION OF ECOLABEL SCORES**

Environmental impact scores for product labels were generated using the ingredient lists available for each product. Information on the ingredients list was used to:

1. identify the relative composition of ingredients (e.g. 10% ingredient X);
2. link each ingredient to a global environmental life cycle assessment database; and
3. calculate the environmental impact per product for four environmental indicators (greenhouse gas emissions, scarcity weighted water stress, land use related biodiversity loss, and eutrophication potential) based on the composition of each ingredient, the type of ingredient (e.g. a mushroom, a tomato, or poultry meat), and environmental information in the life cycle assessment database.
4. create an overall score from percentile scores for each indicator, whereby a score of 1 represents the option with the lowest environmental impact, while a score of 100 represents options with the highest environmental impact. The overall environmental impact score for options is calculated by taking the mean percentile across the four indicators.

Label scores of A were awarded to options falling within the lowest mean environmental impact quintile (i.e. 1^st^ -20^th^ percentiles); scores of B for products in the next quintile (21^st^ -40^th^ percentiles); and so on, with scores of E given to products within the highest mean environmental impact quintile (81^st^-100^th^ percentiles).

More information on the derivation of the environmental impact scores is described in detail elsewhere (Clark et al., 2021).

### **APPENDIX B: POSTER DISPLAYED IN WORKSITES EXPLAINING ECOLABELS**

**
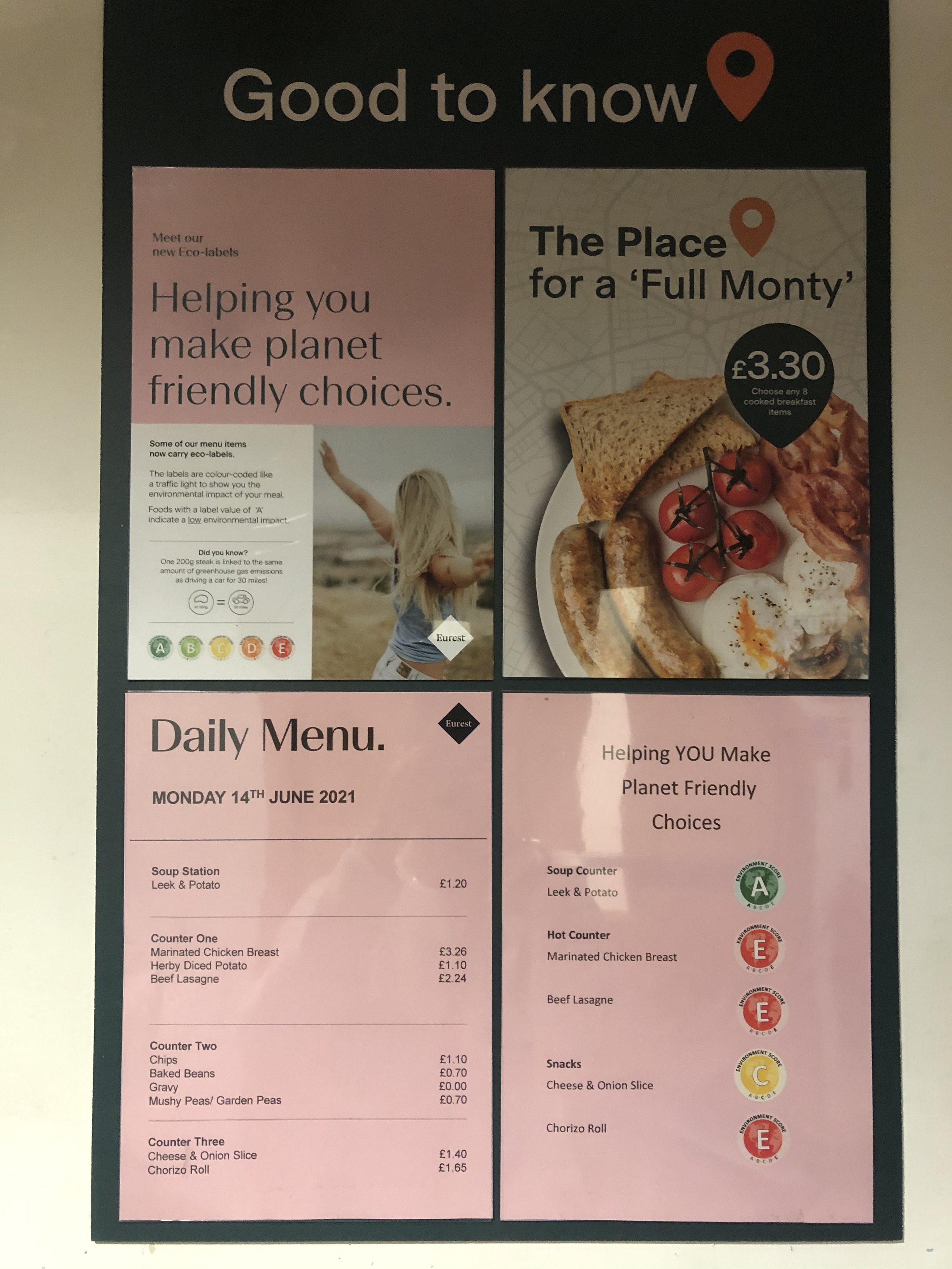
**

**Figures S1a and S1b**. Mean weekly EcoScore (y axis) by study week (x axis), for each intervention site (Figure S1a) and control site (Figure S1b). Vertical lines indicate the start of the trial period during week 15. Numbers above figures indicate site ID numbers.

**Figure S1a** – Intervention sites


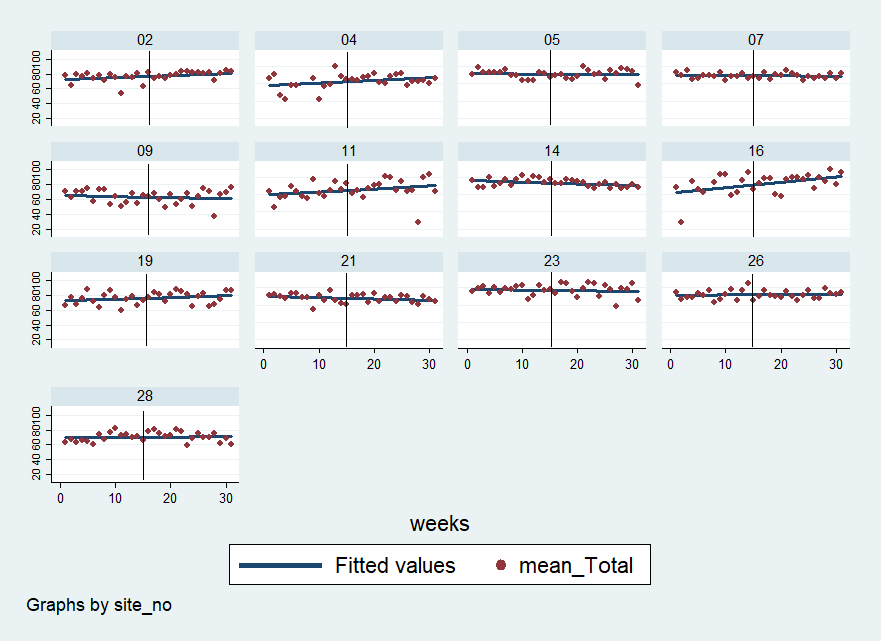


**Figure S1b** – Control sites


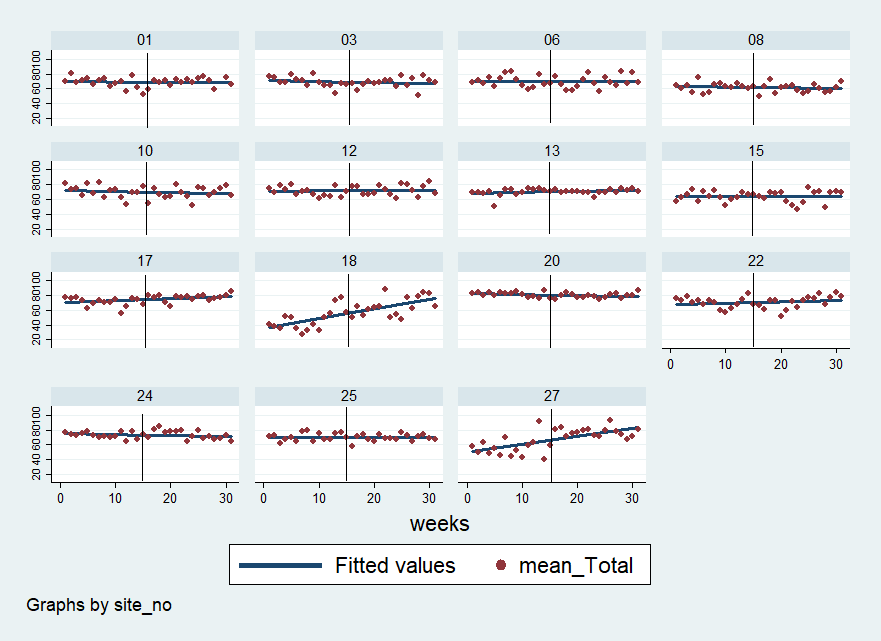


**Table S2**. Regression coefficients (95%CIs) predicting mean weekly EcoScore in per protocol analyses: Including only sites with the highest fidelity (7 intervention sites excluded) vs. excluding sites with the lowest fidelity (1 intervention site excluded)

|  | **Highest fidelity included** | | **Lowest fidelity excluded** | |
| --- | --- | --- | --- | --- |
|  | Coefficients (95%CIs) | p-value | Coefficients (95%CIs) | p-value |
| Intervention group [ref: Control] | 0.46  (-4.83, 5.76) | 0.864 | 1.72  (-2.27, 5.72) | 0.398 |
| Intervention implemented [ref: No] | 1.02  (-1.66, 3.70) | 0.456 | -0.69  (-2.67, 1.30) | 0.497 |
| Week number | 0.16  (0.08, 0.24) | <0.001 | 0.15  (0.07, 0.22) | <0.001 |
| Bank holiday week | -0.06  (-1.77, 1.65) | 0.946 | -0.24  (-1.69, 1.22) | 0.749 |
| Constant | 66.65  (63.62, 69.68) | <0.001 | 66.86  (64.01, 69.71) | <0.001 |
| N | *646 observations from 21 cafeterias (15 control and 6 intervention sites)* | | *832 observations from 27 cafeterias (15 control and 12 intervention sites)* | |

**Table S3**. Mean percentage of meal options available each day by label value

*Calculations assume each available option is purchased at least once. If an option was available but not sold on a particular day, this was not recorded in the purchase data, and therefore not included in this table.*

|  | **Control sites** | | **Intervention sites** | |
| --- | --- | --- | --- | --- |
|  | ***Baseline period*** | ***Intervention period*** | ***Baseline period*** | ***Intervention period*** |
| Percentage of meals scoring A | 28.7 | 29.0 | 26.7 | 27.8 |
| Percentage of meals scoring B | 5.9 | 3.9 | 7.4 | 4.8 |
| Percentage of meals scoring C | 21.6 | 21.2 | 18.5 | 18.7 |
| Percentage of meals scoring D | 15.8 | 15.6 | 17.2 | 15.9 |
| Percentage of meals scoring E | 28.0 | 30.3 | 30.2 | 32.8 |
